# Is It Time for a Requiem for Creatine Supplementation-Induced Kidney Failure? A Narrative Review

**DOI:** 10.3390/nu15061466

**Published:** 2023-03-18

**Authors:** Igor Longobardi, Bruno Gualano, Antonio Carlos Seguro, Hamilton Roschel

**Affiliations:** 1 Applied Physiology and Nutrition Research Group, School of Physical Education and Sport, School of Medicine, University of Sao Paulo, Sao Paulo 01246-903, SP, Brazil; i.long@usp.br (I.L.); gualano@usp.br (B.G.); 2 Rheumatology Division, Hospital das Clinicas HCFMUSP, Faculdade de Medicina, Universidade de São Paulo, Sao Paulo 01246-903, SP, Brazil; 3 Nephrology Division, Hospital das Clinicas HCFMUSP, Faculdade de Medicina, Universidade de São Paulo, Sao Paulo 01246-903, SP, Brazil; trulu@usp.br

**Keywords:** phosphorylcreatine, dietary supplement, glomerular filtration rate, renal function, kidney disease

## Abstract

Creatine has become one of the most popular dietary supplements among a wide range of healthy and clinical populations. However, its potential adverse effects on kidney health are still a matter of concern. This is a narrative review of the effects of creatine supplementation on kidney function. Despite a few case reports and animal studies suggesting that creatine may impair kidney function, clinical trials with controlled designs do not support this claim. Creatine supplementation may increase serum creatinine (Crn) concentration for some individuals, but it does not necessarily indicate kidney dysfunction, as creatine is spontaneously converted into Crn. Based on studies assessing kidney function using reliable methods, creatine supplements have been shown to be safe for human consumption. Further studies with people who have pre-existing kidney disease remain necessary.

## 1. Introduction

Creatine (α-methyl-guanidine-acetic acid) is one of the most popular dietary supplements, with a wide spectrum of potential applications [1]. Creatine is a central player of the phosphagen system, which is crucial for cellular bioenergetics, especially in tissues with high and fluctuating energy demands [2]. Its oral administration can increase muscle creatine content [3], with consistent evidence showing that creatine supplementation can benefit performance in certain sports [4] and improve some clinical symptoms, for instance in rheumatic diseases [5,6], metabolic disturbances [7,8], myopathies [9,10], neurodegenerative diseases [11], chronic obstructive pulmonary disease [12], and congestive heart failure [13,14].

Nonetheless, the potential adverse effects of creatine, particularly on kidney, are still a matter of debate. The first concerns regarding the possible harms of creatine supplementation to kidney came from a series of case studies that have retrospectively associated its use with kidney conditions [15,16,17,18,19,20,21]. In addition, some animal studies also cast doubt on the safety of creatine supplementation [22,23,24]. On the other hand, a growing number of randomized controlled trials, mostly involving healthy individuals, has not found detrimental effects of this supplement on kidney function.

In this narrative review, we discuss the state-of-the-art regarding the impact of creatine supplementation on kidney health, describe gaps in the literature, and provide evidence-based recommendations on creatine-safe consumption.

## 2. The Kidneys and Creatine Metabolism: A Brief Overview

Kidneys are vital organs that are actively involved in several physiological processes, playing a central role in maintaining the body’s homeostasis. They participate in endocrine pathways; regulate osmolarity, extracellular fluid volume, and blood pressure; maintain electrolyte and acid-base balance; and excrete wastes and foreign substances (e.g., toxins and drugs) [25].

Each kidney is formed by 0.8–1.2 million nephrons [26]. In turn, each nephron consists of a glomerulus (a ball-like cluster of capillaries) and a renal tubule (consisting of Bowman’s capsule, proximal and distal convoluted tubules, loop of Henle, and collecting duct) which is formed by a single layer of epithelial cells that regulates urine volume and osmolarity [26]. Initially, plasma is filtered from the glomerulus into Bowman’s capsule. Then subsequent segments of the renal tubule modify the filtered fluid before its excretion as urine. Water, electrolytes (e.g., Na^+^, Ca^2+^, Cl^−^), and some organic compounds (e.g., glucose, amino acids, etc.) can be reabsorbed from the renal tubule into blood according to the individual’s needs, whereas K^+^, other organic compounds (e.g., urea, citric acid cycle intermediates, etc.), and xenobiotics (e.g., penicillin) are secreted from the extracellular fluid into the renal tubule [25,27,28]. All these processes are specifically and strictly controlled through local and/or systemic mechanisms.

In particular, the filtration process mainly depends on capillary pressure and permeability of the glomerular filtration barrier, which is relatively selective and quite robust. Three filtration barriers (fenestrated endothelial cells, the glomerular basement membrane, and specialized cells called podocytes) allow most plasma components to enter the tubule lumen, apart from blood cells and large plasma proteins (e.g., albumin), which are retained and flow into the peritubular capillaries [25]. The leak of such macromolecules into the urine is a strong indicator of disruption of the glomerular permeability [29,30]. Importantly, the kidneys have a substantial reserve capacity, and kidney function is a dynamic process that is continuously adjusted through changes in the internal environment. It is estimated that at least three-quarters of the kidneys’ functional capacity must be lost before homeostasis begins to be severely affected [31].

Glomerular filtration rate (i.e., the amount of fluid that filters into Bowman’s capsule per unit time) is considered a central point in the evaluation of kidney function. Although it cannot be directly measured, glomerular filtration rate can be inferred from the clearance of a filtered solute. The gold-standard method for its assessment is the measure of exogenous filtration biomarkers (i.e., measured glomerular filtration rate; mGFR), which are continuously infused intravenously from both blood and timed urine samples [32]. Because these biomarkers are not produced by the organism (e.g., inulin, ^99m^Tc-DTPA, ^51^Cr-EDTA etc.), it can be assumed that their clearance is equal to the glomerular filtration rate as they are freely filtered but neither reabsorbed nor secreted by the renal tubule. Unfortunately, mGFR is generally limited to specialized facilities so that in medical practice glomerular filtration rates are usually estimated from the serum levels of endogenous biomarkers (i.e., estimated glomerular filtration rate; eGFR), without requiring a direct clearance measurement [32]. For instance, serum levels of creatinine (Crn) and Cystatin C (CysC) have been associated with kidney function and the progression of kidney diseases [33,34]. However, eGFR is directly affected by the rate at which such biochemical markers are produced by metabolic processes, their tubular secretion and reabsorption, and their excretion. Despite major advances in recent years, there are still many sources of bias and errors associated with eGFR methods that may enhance their inaccuracy (e.g., smoking, inflammation, medications, levels of thyroid and corticosteroid hormones, adiposity, muscle mass, diet), which may limit their ability to accurately assess kidney function for certain groups [32,35].

**Figure 1:**
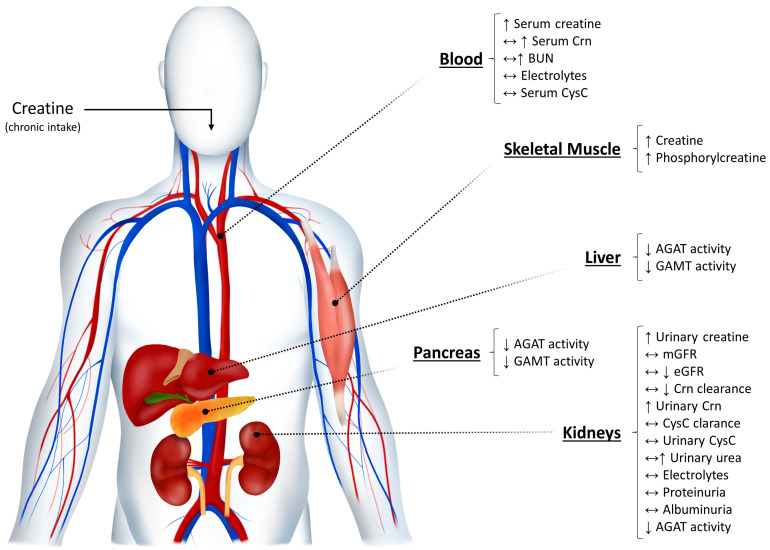
Effects of creatine supplementation on creatine metabolism and kidney function parameters, based on randomized controlled trials in humans [5,38,40,41,42,43,44,45,46,47,48,49,50,51,52,53,54,55,56,57,58,59,60,61,62,63,64,65,66,67,68]. Oral intake of creatine increases total creatine body pool (particularly in skeletal muscle), also leading to an increase (in some cases) in serum Crn from the spontaneous (i.e., non-enzymatically) and irreversible degradation of creatine into Crn, at a rate of ~2% of the total body pool per day. At the same time, AGAT activity is inhibited in the liver, pancreas, and kidneys, decreasing the formation of guanidinoacetate and the endogenous creatine biosynthesis by GAMT. In response to supplementation, creatine’s amino acid precursors could be deviated to alternative metabolic pathways (e.g., the urea cycle), potentially increasing plasma levels of blood nitrogen compounds (e.g., BUN) and its urinary excretion. Although natural fluctuations in Crn may occasionally occur due to creatine intake, kidney function remains preserved as concluded from the stable levels of several biomarkers independent of creatine metabolism (e.g., mGFR, CysC, proteinuria, albuminuria, etc.). AGAT: L-arginine:glycine amidinotransferase; BUN: blood urea nitrogen; Crn: creatinine; CysC: cystatin C; eGFR: estimated glomerular filtration rate; GAMT: guanidinoacetate methyltransferase; mGFR: measured glomerular filtration rate. Arrows indicate the direction of change of a given parameter following creatine supplementation; i.e., increased (↑), decreased (↓), and/or unchanged (↔).

This is the case of individuals taking creatine supplementation. Serum Crn is the most commonly used parameter to assess kidney function, either by itself or as a mean to estimate glomerular filtration rate [34,35]. Crn is an end product of creatine metabolism [36]. Creatine is spontaneously (i.e., non-enzymatically) and irreversibly degraded to Crn at a rate of approximately 2% of the total body pool per day [36]. As chronic creatine intake enhances total body creatine content, it is plausible that a physiological surge of Crn may occur in the blood after creatine administration without necessarily implying any harm to the kidneys (Figure 1). It is worth noting that the opposite also holds true. Vegetarians present with lower serum Crn and Crn clearance, owing to low dietary creatine intake [37]. Accordingly, creatine intake is a known confounder of Crn levels. This is the reason why Crn clearance calculated from equations that consider only serum Crn (i.e., without accounting for its concentration in urine) could be inadequate for those consuming creatine supplements. Whenever this bias is overlooked, it can lead to misinterpretation of tests results and an incorrect diagnosis (i.e., false positive). In addition, it has been suggested that exogenous creatine intake inhibits the L-arginine:glycine amidinotransferase (AGAT) reaction, a crucial step in endogenous de novo creatine biosynthesis in humans [38]. Even though it may favor a balance between creatine intake and its degradation, the recommended dosages used in creatine supplementation protocols typically exceed both endogenous production and excretion rates. Furthermore, the suppression of AGAT activity could also enhance the utilization of creatine’s amino acid precursors by other metabolic pathways (e.g., urea cycle, guanidine cycle) [36,38,39]. Nonetheless, there is currently limited evidence to suggest that this may lead to a significant rise in plasma and urinary levels of urea in healthy individuals.

The overall implication is that any serum biomarker that can be influenced by creatine metabolism may not be sufficiently accurate to assess kidney function in individuals consuming creatine supplements. In this case, whenever mGFR is unfeasible, it is advisable that multiple markers of kidney function should be assessed. The assessments of the urinary content of blood cells (i.e., hematuria), albumin (i.e., albuminuria), proteins (i.e., proteinuria), and other plasma substances not usually found in urine may provide valuable information regarding eventual changes in glomerular membrane permeability and overall kidney health [29,30].

## 3. Creatine Supplementation and Kidney Function

### 3.1. Evidence from Animal Models

Evidence from experimental studies using animal models is summarized in Table 1. Edmunds et al. (2001) conducted a randomized controlled study using Han:Sprague Dawley Cy rats, a well-accepted animal model of inherited renal cystic disease, to investigate the effects of creatine supplementation on kidney parameters [22]. The animals were fed either a standard purified diet for laboratory rodents or the same diet supplemented with creatine/glutamine mixture (5:1 *w*/*w*). Specifically, creatine was administered at a loading dose of 2.0 g∙kg^−1^ of diet for the first week, followed by 5 weeks during which the dose was a fifth of this amount. Creatine supplementation increased serum Crn (only in males), reduced Crn clearance, and enhanced blood urea nitrogen, suggesting an impaired kidney function [22]. The authors also reported greater cyst growth in creatine-supplemented animals compared to controls [22]. These results indicated that creatine could potentially exacerbate renal deterioration in pre-existing kidney conditions.

Taes et al. (2003) also investigated the chronic effects of creatine supplementation on pre-existing kidney failure using nephrectomized male Wistar (two-thirds nephrectomy) and sham-operated rats [69]. Animals were fed either a standard chow or standard chow supplemented with creatine monohydrate (2% *w*/*w*) for 4 weeks. The nephrectomized animals presented with a moderate kidney failure compared to sham-operated ones; but, in contrast to the results of the previously mentioned study [22], creatine supplementation did not further harm kidney function [69]. Importantly, ablation-induced kidney dysfunction in previously healthy animals does not actually correspond to an intrinsic kidney disease. Thus, one may conjecture that kidney function may be differentially affected by creatine supplementation across different experimental models.

Ferreira et al. (2005) had male Wistar rats receiving a standard chow supplemented or not with creatine monohydrate (2 g∙kg^−1^∙day^−1^ of diet) in combination or not with aerobic treadmill exercise for 10 weeks [23], whereas Souza et al. (2009) had male Wistar rats receiving either a control diet or the same diet supplemented with creatine monohydrate (1–5 g∙kg^−1^∙day^−1^ of diet), associated or not with swimming training for 8 weeks [24]. In both studies, creatine-induced deterioration in kidney function biomarkers (including mGFR) was only observed in the non-exercised animals, indicating that exercise may have a protective effect over kidney function in creatine-supplemented animals.

Although experimental models suggest that creatine supplementation could impair kidney parameters, it should be remembered that data obtained in animals often cannot be extrapolated to humans. From an evolutionary perspective, this is particularly true for creatine studies, because the presence of this nutrient in the diet varies largely across species. This may explain why creatine is fully absorbed by humans [3], while it is not bioavailable at all in horses [70], for example. In fact, even close species may respond differently to creatine intake. For instance, Tarnopolsky et al. (2003) observed that creatine supplementation (2% *w*/*w*) induced hepatitis in SOD1 G93A transgenic mice and CD-1 non-transgenic strains of mice, but not in Sprague-Dawley rats, suggesting that a large inter-species variability should be expected following creatine intake [71]. Accordingly, conclusions drawn from animal studies assessing creatine are limited, and prompt generalizations to humans should be avoided.

### 3.2. Evidence from Case-Studies

There have been a number of case studies (Table 2) associating creatine supplementation with kidney dysfunction [15,16,17,18,19,20,21,72]. Nonetheless, as discussed below, many of them have severe limitations that undermine their conclusions.

Pritchard & Kalra (1998) were the first to associate creatine supplementation with kidney dysfunction [15]. They reported the case of a 25-year-old man undergoing a pre-season soccer camp who presented with lower mGFR, higher serum Crn, and lower Crn clearance, supposedly after taking creatine supplements for the previous 8 weeks within recommended dosages. The authors concluded to have “strong circumstantial evidence that creatine was responsible for the deterioration in kidney function in this case” [15]. However, it is noteworthy that the patient already had a pre-existing kidney condition (focal segmental glomerulosclerosis with frequently relapsing nephrotic syndrome) and had also been chronically using cyclosporin, a nephrotoxic medication [73], for the past 5 years.

Koshy et al. (1999) reported the case of a 20-year-old healthy man who developed acute focal interstitial nephritis [16]. Even though the authors attributed the kidney condition to the use of creatine monohydrate supplements (20 g∙day^−1^ for 4 weeks), they did not provide further information regarding the clinical and/or training history of their patient, which is of obvious relevance (see below).

Several cases studies involve bodybuilders. Robinson (2000) reported the case of a 24-year-old bodybuilder who had been using creatine (25 g∙day^−1^ for 12 months) and presented with acute quadriceps compartment syndrome and rhabdomyolysis, causing proteinuria and hematuria [17]. Importantly, the patient participated in approximately 3 h of lower-limb training on the day before his admission to the emergency department, which may have mainly contributed to the episode. Révai et al. (2003) reported the case of a 22-year-old bodybuilder who had been continuously using 200 g∙day^−1^ of creatine along with methandion (an anabolic steroid) and presented with membranoproliferative glomerulonephritis [19]. For obvious reasons, the unusual large amounts of creatine (assuming this is actually accurate) combined with the abusive use of anabolic steroid preclude any assertive conclusions. Thorsteinsdottir et al. (2006) reported the case of a 24-year-old bodybuilder who presented with acute renal failure and acute interstitial nephritis [20]. It is worth noting that this patient had been using creatine monohydrate within recommended dosages along with large amounts of numerous other dietary supplements for bodybuilding purposes (e.g., amino acids, multiple herbal and non-herbal supplements, and vitamins) and had an unusual high-intensity training regimen (3 h of strenuous exercise 5 times per week), which altogether limits the assumptions of the nephrotoxic effects of creatine per se. Taner et al. (2011) reported the case of an 18-year-old bodybuilder who presented with acute tubular necrosis after taking creatine monohydrate within recommended dosages for approximately 7 weeks [21]. However, the authors acknowledge that no previous study had associated creatine supplementation with such a condition. It is more likely that all these cases are related to abnormal exercise training regimens [74] and misuse/abusive use of other substances (including anabolic steroids) [75] rather than to creatine supplementation. Furthermore, a major limitation presented by all studies described above is their retrospective designs, which are mainly biased by self-reporting (e.g., the individual may omit or be imprecise in the information he/she provides to the researchers in relation to training, diet, supplements, drugs, etc.) and lack of “pre-intervention” data (e.g., kidney function might be altered before the individual decided to take creatine).

Two case studies, however, did adopt prospective designs. First, Barisic et al. (2002) reported the case of an 18-year-old sedentary man with mitochondrial encephalopathy and moderate renal insufficiency supplemented with creatine monohydrate (20 g∙day^−1^ for 12 days followed by 5 g∙day^−1^ for 28 months) [18]. Owing to his condition, the patient concomitantly used other medications and dietary supplements along with creatine. Over the course of the treatment, the patient exhibited several alterations in kidney function biomarkers, but the authors speculate that the deterioration occurred as a result of the natural course of his diseases rather than a consequence of creatine supplementation [18]. Gualano et al. (2010) prospectively reported the case of a 20-year-old man with a single kidney undergoing a resistance training program and a high-protein diet (2.8 g∙kg^−1^∙day^−1^), and receiving creatine monohydrate (20 g∙day^−1^ for 5 days followed by 5 g∙day^−1^ for 1 month) [72]. Of note, this study provided information regarding both creatine form and purity, which is important considering that unapproved pharmaceutical ingredients have been detected in several marketed dietary supplements [76]. Despite a decreased mGFR at baseline (which is common to those with a single kidney), no impairment was observed in the athlete’s kidney function [72]. The authors emphasized that the evaluation of serum Crn levels alone could have falsely suggested kidney dysfunction, since the rise in Crn was not accompanied by changes in other parameters, such as mGFR and proteinuria [72].

Although case studies, in general, have the merit of generating new hypotheses, they are surrounded by biases that substantially limit definitive conclusions. Therefore, data from case studies should be treated with extra caution, as they do not support the establishment of causation.

### 3.3. Evidence from Controlled Studies in Humans

Several longitudinal, controlled studies in humans investigated the effects of creatine supplementation in kidney function among different populations [5,38,40,41,42,43,44,45,46,47,48,49,50,51,52,53,54,55,56,57,58,59,60,61,62,63,64,65,66,67,68]. To provide a comprehensive overview, Table 3 and Table 4 summarize the main results of studies conducted in both healthy and clinical populations, respectively.

Poortmans et al. (1997) were one of the pioneers in the search for systematic methods for the evaluation of kidney function in individuals supplemented with creatine. They carried out a non-randomized crossover trial in which five healthy young men received 20 g∙day^−1^ of creatine monohydrate or placebo for 5 days, with an interval of two weeks between conditions [41]. eGFR, serum and urinary Crn, proteinuria, and albuminuria were determined after each experimental session. All markers remained within normal range after creatine intake or control [41]. Poortmans et al. (2005) also evaluated pre-to-post changes in serum Crn, urinary Crn, and albuminuria in 20 healthy young men undergoing creatine monohydrate supplementation (21 g∙day^−1^); no signs of renal impairment were found after 14 days [51]. Poortmans & Francaux (1998) also had 20 healthy young men receiving either placebo or creatine monohydrate supplementation for a longer period of time. No between-group differences were observed in Crn clearance, urea clearance, or albuminuria after 21 g∙day^−1^ for 5 days followed by 3 g∙day^−1^ for 58 days [42]. Poortmans & Francaux (1999) compared nine athletes of national and international levels who had been regularly using creatine monohydrate (1–80 g∙day^−1^ for the past 10–60 months) vs. physical education and physical therapy students who were not users of creatine supplements; no significant between-group differences were detected [43].

Other independent groups also investigated the effects of creatine supplementation on kidney function in athletes. Mayhew et al. (2002) assigned 23 NCAA Division II American football players, with at least 2 years of strength training experience, into either a creatine-supplemented or a control group not receiving any supplements [46]. The average regular daily consumption of creatine ranged from 5 to 20 g∙day^−1^ for 0.25 to 5.6 years. There were no long-term detrimental effects of creatine monohydrate supplementation on kidney function [46]. Kreider et al. (2003) evaluated the long-term effects of creatine supplementation on the clinical biomarkers of kidney function in 116 football players from National Collegiate Athletic Association Division IA [49]. In an open label manner, athletes chose whether they wanted to take creatine monohydrate or non-creatine containing supplements during their regular training regimen over a 2-year period. The mean creatine usage was 15.75 g∙day^−1^ for 5 days, followed by 5–10 g∙day^−1^ for 0–21 months. Participants were categorized as (i) non-creatine users, (ii) subjects who ingested creatine for 0–6 months, (iii) subjects who ingested creatine for 7–12 months, and (iv) subjects who ingested creatine for 12–21 months. No clinically significant alterations in any marker were found, suggesting that short and long-term creatine supplementation (up to 21 months) does not appear to adversely affect kidney function [49]. These studies, however, are limited by their experimental designs, which lack randomization.

In a randomized placebo-controlled trial, Robinson et al. (2000) had 48 healthy young men and women receiving either a creatine monohydrate or placebo, using loading (20 g∙day^−1^ for 5 days) or maintenance (3 g∙day^−1^ for 8 weeks) protocols, in combination or not with resistance training [45]. No impairment in kidney function was found in any group. Similarly, Eijnde et al. (2003) found no change in serum Crn, urinary Crn, and blood urea nitrogen in older men supplemented with creatine monohydrate (5 g∙day^−1^) in combination with a 12-month exercise training program [48]. Conversely, Brose et al. (2003) found increased serum, but not urinary Crn, in resistance training healthy older adults supplemented with creatine monohydrate (5 g∙day^−1^ for 14 weeks) when compared to their control peers receiving placebo [47]. Equivalent results were found by Gualano et al. (2008), who assessed healthy sedentary men undergoing aerobic training supplemented with either creatine monohydrate or placebo (0.3 g∙kg^−1^∙day^−1^ for 1 week followed by 0.15 g∙kg^−1^∙day^−1^ for 11 weeks) [54]. Despite the increased serum Crn in the creatine group, other kidney function biomarkers (serum CysC, serum electrolytes, and urinary electrolytes) remained unaltered. These results re-emphasize the importance of using non-Crn related biomarkers when monitoring creatine users.

In a double-blind, randomized controlled trial, Earnest et al. (1996) investigated the effects of creatine supplementation on middle-aged adults with hypercholesterolemia [40]. The participants received either creatine monohydrate or placebo at a dose of 20 g∙day^−1^ for 5 days, followed by 10 g∙day^−1^ for 51 days. Despite no significant changes being observed in serum Crn levels, the authors reported a significant increase in blood urea nitrogen levels in women, but not in men, who received creatine monohydrate at only one out of the four time-points of sampling. The authors acknowledge that changes in oestrogen status and menstrual cycle timing could also be responsible for the observed changes [40]. Bender et al. (2008) randomly assigned patients with Parkinson’s disease to creatine monohydrate or placebo (20 g∙day^−1^ for 6 days, followed by 2–4 g∙day^−1^ for 6–24 months) in a double-blind fashion [53]. Notably, except for an increase in serum Crn (detected at only one out of the seven time-points of sampling), no other changes were detected in any parameters (e.g., serum CysC and blood urea nitrogen, hematuria, and albuminuria). Corroborating these findings, long-term creatine monohydrate supplementation (10 g∙day^−1^ for 310 days) in patients with amyotrophic lateral sclerosis did not result in increased blood urea nitrogen or albuminuria when compared to placebo. Serum Crn was also similar between groups when assessed via high performance liquid chromatography, but not via enzymatic methods [50]. Collectively, these studies indicate that these fluctuations in biochemical parameters may be, at least partially, attributed to factors related to sex, temporal variation, and methods of assessment.

Our laboratory carried out several studies investigating the impact of creatine supplementation (~5 to 20 g∙day^−1^ up to 24 months) in different populations using multiple biomarkers, including ^51^Cr-EDTA clearance (one of the gold-standard techniques to assess mGFR) in some studies. Overall, we found no evidence of the detrimental effects of creatine monohydrate on kidney function in resistance-trained healthy males consuming high-protein diet (1.2–3.1 g∙kg^−1^∙day^−1^) [59], postmenopausal women with knee osteoarthritis [6], postmenopausal women with osteopenia [63,67], women with fibromyalgia [5], or pre-frail and frail older adults [68].

We also evaluated groups with or at higher risk of developing kidney diseases. We randomly assigned older adults with type 2 diabetes mellitus to either creatine monohydrate or placebo supplementation (5 g∙day^−1^ for 12 weeks), and all of them undertook a combined aerobic and resistance training program [56]. mGFR was assessed through ^51^Cr-EDTA clearance. Blood samples and 24-h urine samples were also obtained. There were no between-group differences for any parameter assessed. In a double-blind, randomized, placebo-controlled study, we administered 0.1 g∙kg^−1^∙day^−1^ of creatine monohydrate to youth with juvenile childhood systemic lupus erythematosus for 12 weeks [60]. Several kidney function parameters were assessed, including ^51^Cr-EDTA clearance. As in the previous studies, no signs of kidney function deterioration were observed.

Altogether, these findings suggest that creatine monohydrate within recommended doses seems to be safe, even when administered in clinical populations. This conclusion is shared by evidence-based position statements [77,78] and further supported by a small meta-analysis that demonstrated that creatine supplementation did not alter serum Crn (five studies included) or blood urea nitrogen levels (six studies included), despite a trend towards lower Crn clearance (three studies included), which might be explained by the already discussed bias inherited by Crn [79]. Indeed, the low number of studies included in the meta-analysis weakens its conclusions.

## 4. Gaps and Recommendations

Although creatine is one of the most studied dietary supplements, relevant gaps exist around its possible effects on kidney function. For instance, there is a scant amount of long-term studies (>16 weeks) evaluating the impact of creatine supplementation on kidney health, particularly using accurate measures. There is also very limited evidence that creatine supplementation is safe for those with pre-existing kidney diseases, a very important limitation in the literature. In addition, considering that a significant part of creatine consumers is composed by amateur and elite athletes who use many other (licit and/or illicit) substances, the impact of “polypharmacy” also involving creatine supplements as a burden to kidney function cannot be disregarded. Additionally, the safety profile of alternative commercialized forms of creatine other than creatine monohydrate cannot be fully stablished because these novel formulations have been much less studied. In fact, one of them, creatine ethyl ester, is a more unstable molecule, favoring increases in serum Crn [55]. Further investigations are necessary to evaluate whether each of these creatine formulations is harmless to kidneys.

Based on these gaps and the evidence available in the literature, it would be advisable that: (i) creatine supplementation should be used in rational doses (up to 20 g∙day^−1^); (ii) those who have a very low glomerular filtration rate induced by a pre-existing kidney disease should refrain from creatine use; (iii) monitoring kidney function is not mandatory for healthy individuals taking creatine, but it should be wise to follow-up those at risk for decreased kidney function (e.g., older individuals and clinical populations) under long-term supplementation protocols; (iv) the assessment of kidney function using markers that are independent of creatine/Crn metabolism is important to avoid misdiagnosis (false positives); (v) the use of new formulations of creatine should be cautionary, unless a safety profile has been scientifically established; and (vi) creatine supplements (generally low-price) with unattested purity and a plethora of contaminants are available in the market and should be avoided, as they may be detrimental to health.

## 5. Conclusions

Despite some anecdotal reports and experimental data suggesting that creatine could be deleterious to the kidneys, cumulative evidence from independent, randomized controlled trials clearly show this is not the case. We did identify some gaps in the literature, based on which we suggest that it is prudent to avoid creatine supplementation for those who have pre-existing kidney diseases resulting in very low kidney functions. For those at risk of decreased glomerular filtration rate (e.g., some older individuals or those with certain clinical conditions), monitoring kidney function while supplementing with creatine appears to be prudent, although large, relatively long-term studies have shown no risks. Finally, consumers should select creatine supplements that have been properly tested and certified for their quality/purity, avoiding the health risks associated with contaminants.

## Figures and Tables

**Table 1:** Experimental studies investigating the effect of creatine supplementation on kidney function in animals.

Study	Sample Characteristics	Experimental Groups	Creatine Supplementation Protocol	Comparison	Main Findings
Edmunds et al. (2001) [22]	Han:SPRD-Cy Rats *	(i) Creatine diet (ii) Control diet	Creatine/glutamine (5:1 *w*/*w*) L: 2.4 g∙kg^−1^∙day^−1^ of diet for 7 days M: 0.48 g∙kg^−1^∙day^−1^ of diet for 35 days	Purified diet	↑ Serum Crn ^#^ ↓ Crn clearance ↑ BUN ↑ Cyst scores
Taes et al. (2003) [69]	Male Wistar rats	(i) Sham-operated/control diet (ii) Nephrectomized/control diet (iii) Sham-operated/creatine diet (iv) Nephrectomized/creatine diet	Creatine monohydrate (2% of diet∙day^−1^) for 4 weeks	Soy-based chow (14% protein)	↔ mGFR ^†^ ↔ Serum Crn ^†^ ↔ Crn clearance ^†^ ↔ BUN ^†^ ↔ Urea clearance ^†^ ↔ Serum CysC ^†^ ↔ Proteinuria ^†^ ↔ Albuminuria ^†^
Ferreira et al. (2005) [23]	Male Wistar rats	(i) Sedentary/control diet (ii) AE/control diet (iii) Sedentary/creatine diet (iv) AE/creatine diet	Creatine monohydrate 2 g∙kg^−1^∙day^−1^ of diet for 10 weeks	Standard chow	↓ mGFR ^&^ ↓ RPF ^&^ ↓ Filtration fraction ^&^ ↔ Proteinuria ↔ UFR
Souza et al. (2009) [24]	Male Wistar rats	(i) Sedentary/control diet (ii) AE/control diet (iii) Sedentary/creatine diet (iv) AE/creatine diet	Creatine monohydrate L: 5 g∙kg^−1^∙day^−1^ of diet for 1 week M: 1 g∙kg^−1^∙day^−1^ of diet for 4–8 weeks	Not specified	↑ Serum Crn ^&^ ↑ BUN ^&^ ↑ RHA ^&^

AE: aerobic exercise; BUN: blood urea nitrogen; Crn: creatinine; CysC: Cystatin C; L: loading phase; M: maintenance phase; mGFR: measured glomerular filtration rate; RHA: renal histological abnormalities; RPF: renal plasma flow; UFR: urine flow rate. * animal model of inherited renal cystic disease; ^#^ only in males; ^†^ significant difference between sham-operated and nephrectomized rats; ^&^ only in non-exercised animals. Arrows indicate similar results (↔) or significant increased (↑)/decreased (↓) in creatine-supplemented animals versus controls.

**Table 2:** Main findings of case studies reporting on creatine supplementation and kidney function.

Study	Study Design	Patient Characteristics	Creatine Supplementation Protocol	Concomitant Use of Other Substances	Main Findings
Pritchard & Kalra (1998) [15]	Retrospective	25-year-old man with FSG and frequently relapsing NS undergoing pre-season soccer training regime	L: 15 g∙day^−1^ for 1 week M: 2 g∙day^−1^ for 7 weeks	Cyclosporine	↓ mGFR ↑ Serum Crn ↓ Crn clearance
Koshy et al. (1999) [16]	Retrospective	20-year-old healthy man	Creatine monohydrate 20 g∙day^−1^ for 4 weeks	None	↑ Serum Crn ↑ Proteinuria ↑ Hematuria
Robinson et al. (2000) [17]	Retrospective	24-year-old healthy man Bodybuilder	25 g∙day^−1^ for 12 months	None	↔ Serum Crn ↔ BUN ↔ Electrolytes ↑ Proteinuria ↑ Hematuria Rhabdomyolysis
Barisic et al. (2002) [18]	Prospective	18-year-old sedentary man with mitochondrial encephalopathy and moderate renal insufficiency	Creatine monohydrate L: 20 g∙day^−1^ for 12 days M: 5 g∙day^−1^ for 28 months	Carbamazepine L-thyroxine Lamotrigine Coenzyme Q Riboflavin Vitamin K3 Ascorbic acid L-carnitine	↑ Serum Crn ↓ Crn clearance ↑ BUN ↑ Proteinuria
Révai et al. (2003) [19]	Retrospective	22-year-old man Supposedly bodybuilder	200 g∙day^−1^ continuously	Methandion	MPGN *
Thorsteinsdottir et al. (2006) [20]	Retrospective	24-year-old man Bodybuilder ^#^	Creatine monohydrate 15 g∙day^−1^ for 6 months	Large amounts of dietary supplements for bodybuilding purposes, including multiple herbs, nonherbal supplements, and vitamins	↑ Serum Crn ↓ Crn clearance ↑ BUN ↑ Proteinuria ↑ Hematuria
Gualano et al. (2010) [72]	Prospective	20-year-old man with a single kidney and mild renal insufficiency submitted to resistance training and a high-protein diet (2.8 g∙kg^−1^∙day^−1^)	Creatine monohydrate L: 20 g∙day^−1^ for 5 days M: 5 g∙day^−1^ for 30 days	None	↔ mGFR ↑ Serum Crn ↓ Crn clearance ↓ BUN ↔ Electrolytes ↔ Proteinuria ↓ Albuminuria
Taner et al. (2010) [21]	Retrospective	18-year-old healthy man Supposedly bodybuilder	Creatine monohydrate L: 20 g∙day^−1^ for 5 days M: 1 g∙day^−1^ for 6 weeks	Not reported	↑ Serum Crn ↑ BUN ↑ Urate ↔ Electrolytes ↑ Proteinuria

BUN: blood urea nitrogen; Crn: creatinine; FSG: focal segmental glomerulosclerosis; L: loading phase; M: maintenance phase; mGFR: measured glomerular filtration rate; MPGN: membranoproliferative glomerulonephritis; NS: nephrotic syndrome. * the diagnosis was provided without any supporting test results being presented; ^#^ unusual high-intensity training regimen (3 h of strenuous exercise five times per week). Arrows indicate measured values above (↑), below (↓), or within (↔) normative range.

**Table 3:** Experimental studies investigating the effects of creatine supplementation on kidney function in healthy populations.

Study	Sample Characteristics	Experimental Design	Creatine Supplementation Protocol	Main Findings
Derave et al. (2004) [38]	Healthy young adults (19 ± 0 years old)	Double-blind randomized controlled trial (i) Creatine (n = 8) (ii) Placebo (n = 8)	Creatine monohydrate L: 20 g∙day^−1^ for 1 week M: 5 g∙day^−1^ 19 weeks	↑ Serum Crn ↔ Urinary Crn ↔ BUN
Kreider et al. (2003) [49]	Healthy young adults (19 ± 2 years old) College football players	Non-randomized controlled trial (i) Non-creatine control (n = 44) (ii) Creatine 0–6 months (n = 12) (iii) Creatine 7–12 months (n = 25) (iv) Creatine 12–21 months (n = 17)	Creatine monohydrate L: 15.75 g∙day^−1^ for 5 days M: 5–10 g∙day^−1^ for 0–21 months	↔ Serum Crn ↔ BUN ↔ Uric acid ↔ Electrolytes ↔ Plasma protein ↔ Plasma albumin ↔ Plasma globulin
Mayhew et al. (2002) [46]	Healthy young adults (20 ± 2 years old) College football players	Non-randomized controlled trial (i) Creatine users (n = 10) (ii) Non-creatine control (n = 13)	Creatine monohydrate 5–20 g∙day^−1^ for 0.25–5.6 years	↔ Serum Crn ↔ Crn clearance ↔ BUN
Spillane et al. (2009) [55]	Healthy young men (20 ± 2 years old)	Double-blind randomized controlled trial (i) Creatine monohydrate + RT (n = 10) (ii) Creatine ethyl ester + RT (n = 10) (iii) Placebo + RT (n = 10)	Creatine monohydrate or Creatine ethyl ester L: 20 g∙day^−1^ for 5 days M: 5 g∙day^−1^ for 43 days	↔↑ Serum Crn ^#^
Cancela et al. (2008) [52]	Healthy young men (20 ± 3 years old) Soccer players	Double-blind randomized controlled trial (i) Creatine (n = 7) (ii) Placebo (n = 7)	Creatine monohydrate L: 15 g∙day^−1^ for 7 days M: 3 g∙day^−1^ 49 days	↔ Serum Crn ↔ Crn clearance ↔ BUN ↔ Uric acid ↔ Plasma albumin
Poortmans & Francaux (1998) [42]	Healthy young men (21 ± 2 years old)	Non-randomized controlled trial (n = 20) (i) Creatine supplementation (ii) Placebo supplementation	Creatine monohydrate L: 21 g∙day^−1^ for 5 days M: 3 g∙day^−1^ for 58 days	↔ Crn clearance ↔ Urea clearance ↔ Albuminuria
Mihic et al. (2000) [44]	Healthy young adults (22 ± 2 years old) Physically active	Randomized controlled trial (i) Creatine (n = 15; 7 men/8 women) (ii) Placebo (n = 15; 8 men/7 women)	Creatine monohydrate 20 g∙day^−1^ for 5 days	↔ Serum Crn ↔ Crn clearance
Poortmans & Francaux (1999) [43]	Healthy young adults (24 ± 3 years old) (i) Athletes of national and international levels (ii) Physical education and physical therapy students	Non-randomized controlled trial (i) Creatine supplemented (n = 9) (ii) Non-creatine control (n = 85)	Creatine monohydrate 1–80 g∙day^−1^ for 10–60 months	↔ Serum Crn ↔ Urinary Crn ↔ Crn clearance ↔ BUN ↔ Urinary urea ↔ Urea clearance ↔ Plasma albumin ↔ Albuminuria ↔ Clearance albumin
Gualano et al. (2008) [54]	Sedentary healthy young men (24 ± 5 years old) *	Double-blind randomized controlled trial (i) Creatine + AT (n = 9) (ii) Placebo + AT (n = 9)	Creatine monohydrate L: 0.3 g∙kg^−1^∙day^−1^ for 1 week M: 0.15 g∙kg^−1^∙day^−1^ for 11 weeks	↑ Serum Crn ↓ Serum CysC ↔ Serum electrolytes ↔ Urinary electrolytes
Carvalho et al. (2011) [58]	Healthy male adults (24 ± 5 years old) *	Double-blind randomized controlled trial (i) Creatine-absolute + RT (n = 12) (ii) Creatine-relative + RT(n = 11) (iii) Placebo + RT (n = 12)	Creatine monohydrate L: 20 g∙day^−1^ for 1 week M: 0.03 g∙kg^−1^ or 5 g∙day^−1^ for 7 weeks	↑ Serum Crn ^†^ ↔ BUN ↔ Proteinuria ↔ Hematuria
Poortmans et al. (2005) [51]	Healthy young men (24 ± 6 years old)	Single group pre-to-post design (i) Creatine supplementation (n = 20)	Creatine monohydrate 21 g∙day^−1^ for 14 days	↔ Serum Crn ↔ Urinary Crn ↔ Albuminuria
Poortmans et al. (1997) [41]	Healthy young men (25 ± 3 years old)	Non-randomized crossover (n = 5) (i) Creatine supplementation (ii) Placebo supplementation	Creatine monohydrate 20 g∙day^−1^ for 5 days	↔ eGFR ↔ Serum Crn ↔ Urinary Crn ↔ Crn clearance ↔ Proteinuria ↔ Albuminuria
Robinson et al. (2000) [45]	Healthy young adults (25 ± 5 years old) * Physically active	Randomized placebo-controlled trial (i) Creatine (L; n = 7 men) (ii) Creatine (L; n = 6 men) ^‡^ (iii) Creatine (M; n = 7 women) (iv) Creatine + RT (M; n = 9 women) (v) Placebo (n = 7 men) (vi) Placebo (n = 6; 3 men/3 women) ^‡^ (vii) Placebo + RT (n = 6 women)	Creatine monohydrate L: 20 g∙day^−1^ for 5 days M: 3 g∙day^−1^ for 8 weeks	↔ Serum Crn ↔ BUN ↔ Electrolytes ↔ Plasma albumin
Lugaresi et al. (2013) [59]	Healthy young men (26 ± 4 years old) * Resistance trained High-protein diet	Double-blind randomized controlled trial (i) Creatine (n = 12) (ii) Placebo (n = 14)	Creatine monohydrate L: 20 g∙day^−1^ for 1 week M: 5 g∙day^−1^ for 11 weeks	↔ mGFR ↔ Serum Crn ↔ BUN ↔ Serum electrolytes ↔ Urinary electrolytes ↔ Proteinuria ↔ Albuminuria
Blancquaert et al. (2018) [65]	Healthy young women (26 ± 7 years old)	Randomized controlled trial (i) Omnivorous diet (Control; n = 10) (ii) Vegetarian diet + Placebo (n = 15) (iii) Vegetarian diet + Supplements (n = 15)	Creatine monohydrate 1 g∙day^−1^ for 3–6 months	↔ Serum Crn ↔ Urinary Crn
Pereira et al. (2015) [64]	Healthy young adults (29 ± 4 years old)	Non-counterbalanced single-blind crossover (i) Creatine (L) (ii) Creatine (M) (iii) Placebo	Creatine monohydrate L: 7 or 20 g∙day^−1^ for 7 days M: 2 or 5 g∙day^−1^ for 23 days	↔ Serum Crn ↔ Urinary Crn
Chilibeck et al. (2015) [61]	Postmenopausal women (57 ± 6 years old)	Double-blind randomized controlled trial (i) Creatine + RT (n = 15) (ii) Placebo + RT (n = 18)	Creatine monohydrate 0.1 g∙kg^−1^∙day^−1^ for 12 months	↔ Serum Crn ↔ Crn clearance ↔ BUN ↔ Plasma albumin ↔ Proteinuria ↔ Albuminuria
Eijnde et al. (2003) [48]	Physically active healthy older men (63 ± 9 years old) *	Double-blind randomized controlled trial (i) Creatine + CT (n = 15) (ii) Placebo + CT (n = 21)	Creatine monohydrate 5 g∙day^−1^ for 12 months	↔ Serum Crn ↔ Urinary Crn ↔ BUN
Brose et al. (2003) [47]	Healthy older adults (68 ± 4 years old) *	Double-blind randomized controlled trial (i) Creatine + RT (n = 14; 8 men/6 women) (ii) Placebo + RT (n = 14; 7 men/7 women)	Creatine monohydrate 5 g∙day^−1^ for 14 weeks	↑ Serum Crn ↔ Urinary Crn

AT: aerobic training; BUN: blood urea nitrogen; Crn: creatinine; CT: combined aerobic and strengthening exercise training; CysC: Cystatin C; eGFR: estimated glomerular filtration rate; L: loading phase; M: maintenance phase; mGFR: measured glomerular filtration rate; RT: resistance training. * whole sample estimated pooled mean ± SD from available data. ^#^ only creatine ethyl ester supplementation significant increased serum Crn levels. ^†^ remained within normal ranges. ^‡^ assessments were performed six weeks after the last ingested dose of dietary supplement. Arrows indicate similar results (↔) between-groups or significant increased (↑)/decreased (↓) in creatine-supplemented groups versus placebo/control.

**Table 4:** Experimental studies investigating the effects of creatine supplementation on kidney function in clinical populations.

Study	Sample Characteristics	Experimental Design	Creatine Supplementation Protocol	Main Findings
Hayashi et al. (2014) [60]	Children with SLE (15 ± 2 years old)	Double-blind randomized placebo-controlled crossover trial (n = 15) (i) Creatine (ii) Placebo	Creatine monohydrate 0.1 g∙kg^−1^∙day^−1^ for 12 weeks	↔ mGFR ↔ Serum Crn ↔ Urinary Crn ↔ BUN ↔ Urinary urea ↔ Serum electrolytes ↔ Proteinuria ↔ Albuminuria
Alves et al. (2013) [5]	Middle-aged women with fibromyalgia (49 ± 9 years old) *	Double-blind randomized controlled trial (i) Creatine (n = 15) (ii) Placebo (n = 13)	Creatine monohydrate L: 20 g∙day^−1^ for 5 days M: 5 g∙day^−1^ for 15 weeks	↔ Serum Crn ↔ Urinary Crn ↔ BUN ↔ Urinary urea ↔ Serum electrolytes ↔ Urinary electrolytes ↔ Proteinuria ↔ Albuminuria
Earnest et al. (1996) [40]	Middle-aged adults with hypercholesterolemia (51 ± 12 years old)	Double-blind randomized controlled trial (i) Creatine (n = 20; 9 men/11 women) (ii) Placebo (n = 14; 9 men/5 women)	Creatine monohydrate L: 20 g∙day^−1^ for 5 days M: 10 g∙day^−1^ for 51 days	↔ Serum Crn ↔↑ BUN ^#^
Gualano et al. (2011) [56]	Sedentary older adults with T2DM (57 ± 6 years old) *	Double-blind randomized controlled trial (i) Creatine + CT (n = 13) (ii) Placebo + CT (n = 12)	Creatine monohydrate 5 g∙day^−1^ for 12 weeks	↔ mGFR ↔ Serum Crn ↔ Urinary Crn ↔ Crn clearance ↔ BUN ↔ Urinary urea ↔ Serum electrolytes ↔ Urinary electrolytes ↔ Proteinuria ↔ Albuminuria
Neves et al. (2011) [57]	Postmenopausal women with knee osteoarthritis (58 ± 3 years old)	Double-blind randomized controlled trial (i) Creatine (n = 13) (ii) Placebo (n = 11)	Creatine monohydrate L: 20 g∙day^−1^ for 1 week M: 5 g∙day^−1^ for 11 weeks	↔ mGFR ↔ Serum Crn ↔ Urinary Crn ↔ Crn clearance ↔ BUN ↔ Urinary urea ↔ Proteinuria ↔ Albuminuria
Lobo et al. (2015) [63]	Postmenopausal women with osteopenia (58 ± 5 years old) *	Double-blind randomized controlled trial (i) Creatine (n = 56) (ii) Placebo (n = 53)	Creatine monohydrate 1 g∙day^−1^ for 12 months	↔ Serum Crn ↔ Urinary Crn ↔ Albuminuria
Sales et al. (2020) [67]	Postmenopausal women with osteopenia (58 ± 6 years old)	Double-blind randomized controlled trial (i) Creatine (n = 106) (ii) Placebo (n = 94)	Creatine monohydrate 3 g∙day^−1^ for 24 months	↔ Serum Crn ↔ Urinary Crn ↔ Albuminuria
Groeneveld et al. (2005) [50]	Young to older adults with ALS (58 ± 11 years old)	Double-blind randomized controlled trial (i) Creatine (n = 88; 57 men/31 women) (ii) Placebo (n = 87; 63 men/24 women)	Creatine monohydrate 10 g∙day^−1^ for 310 days	↔↑ Serum Crn ^†^ ↔ BUN ↔ Albuminuria
Bender et al. (2008) [53]	Middle-aged patients with Parkinson (60 ± 10 years old)^*^	Double-blind randomized controlled trial (i) Creatine (n = 40; 28 men/12 women) (ii) Placebo (n = 20; 15 men/5 women)	Creatine monohydrate L: 20 g∙day^−1^ for 6 days M: 2–4 g∙day^−1^ for 6–24 months	↔↑ Serum Crn ^‡^ ↔ Urinary Crn ↔ Serum CysC ↔ BUN ↔ Hematuria ↔ Albuminuria
Kieburtz et al. (2015) [62]	Middle-aged and older adults with Parkinson (62 ± 10 years old) *	Double-blind randomized controlled trial (i) Creatine (n = 477) (ii) Placebo (n = 478)	Creatine monohydrate 10 g∙day^−1^ for 5–8 years	↔↑ Serum Crn ^¥^
Domingues et al. (2020) [66]	Middle-aged and older adults with peripheral arterial disease (64 ± 9 years old) *	Double-blind randomized controlled trial (i) Creatine (n = 14) (ii) Placebo (n = 15)	Creatine monohydrate L: 20 g∙day^−1^ for 1 week M: 5 g∙day^−1^ for 7 weeks	↔ Serum Crn ↔ Urinary Crn ↔ Crn clearance
Roschel et al. (2021) [68]	Pre-frail and frail older adults (72 ± 6 years old)	Double-blind randomized controlled trial (i) Creatine (n = 22) (ii) Creatine + Whey (n = 22) (iii) Whey (n = 22) (iv) Placebo (n = 22)	Creatine monohydrate 6 g∙day^−1^ for 16 weeks	↔ mGFR ↔ Serum Crn ↔ Urinary Crn ↔ BUN ↔ Urinary urea ↔ Proteinuria ↔ Albuminuria

ALS: amyotrophic lateral sclerosis; BUN: blood urea nitrogen; Crn: creatinine; CT: combined aerobic and strengthening exercise training; CysC: Cystatin C; eGFR: estimated glomerular filtration rate; L: loading phase; M: maintenance phase; mGFR: measured glomerular filtration rate; SLE: systemic lupus erythematosus; T2DM: type 2 diabetes mellitus. * whole sample estimated pooled mean ± SD from available data. ^#^ significantly increased at one of four time-points in women, but not in men. ^†^increased only when assessed by enzymatic methods, but not with HPLC (High Performance Liquid Chromatography). ^‡^ between-group significant difference detected at only one out of the seven time-points of sampling. ^¥^ heat maps by treatment group showed an immediate increase in serum Crn levels at the first post-baseline visit followed by stabilization in the creatine group. Arrows indicate similar results (↔) between-groups or significant increased (↑)/decreased (↓) in creatine-supplemented groups versus placebo/control.

## Data Availability

Not applicable.
